# Supplementary material for: Enhanced anti-tumour immunity requires the interplay between resident and circulating memory CD8+ T cells
Source: Nat Commun. 2017 Jul 17;8:16073. doi: 10.1038/ncomms16073 (PMC5520051; doi:10.1038/ncomms16073)
Supplement: Supplementary Information [file ncomms16073-s1.pdf]

File Name: Supplementary Information
Description: Supplementary Figures

File Name: Peer Review File
Description:

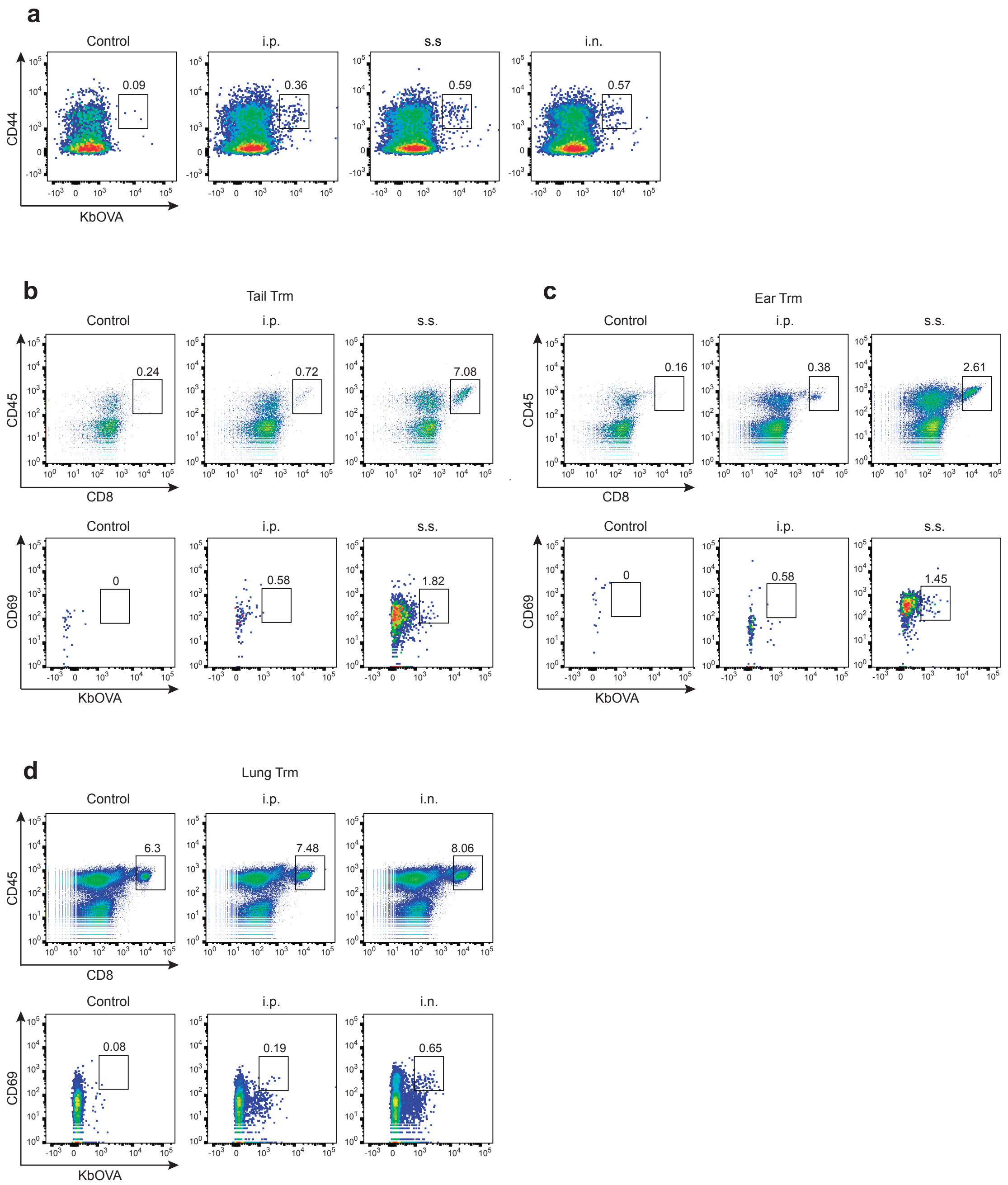

Supplementary Figure 1. Generation of Trm cells after different routes of rVACV-OVA infection (**a**) Representative FACS dot-plots of endogenous OVA-specific circulating memory CD8$^+$ T cells in the dLN 30 d after i.p. (5×10^4 p.f.u.), s.s. (2×10^6 p.f.u.) or i.n. (5×10^4 p.f.u.) infection with rVACV-OVA. (**b-d**) Representative FACS dot-plots showing endogenous CD45$^+$CD8$^+$ cells (top panels), and OVA-specific CD69$^+$ Trm cells (bottom panels) infiltrating the tail skin (**b**) or the ear (**c**) 30 d after s.s. in the tail, and in the lung (**d**) 30 d after i.n. infection with rVACV-OVA.

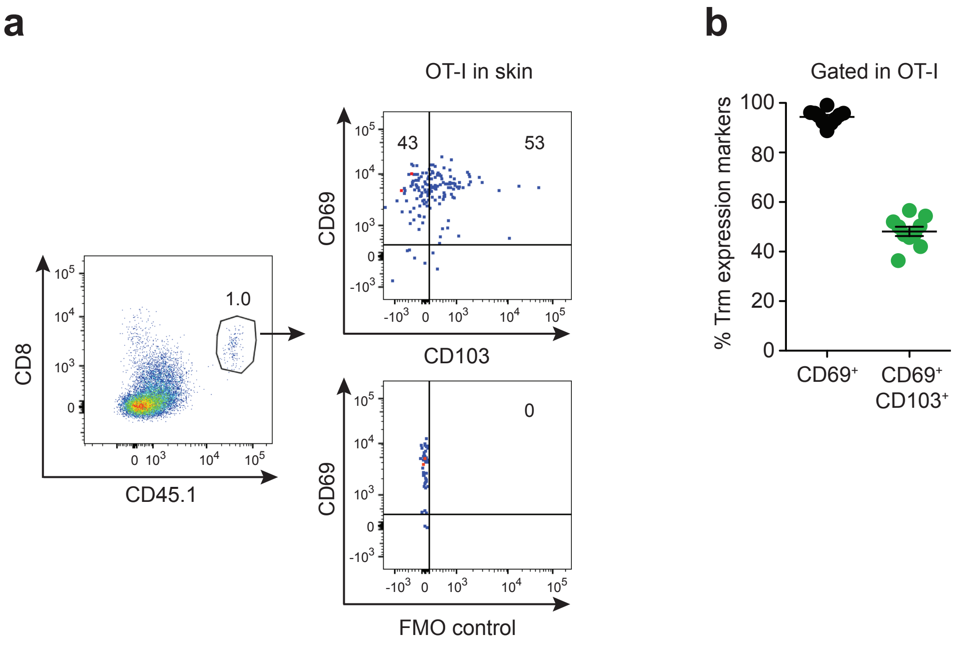

Supplementary Figure 2. Tcm cells give rise to Trm cells upon MC38-OVA inoculation
(**a**) Representative FACS dot-plots for OT-I identification (left) and Trm expression markers (right). (**b**) Frequency of Trm expression markers in OT-I cells in the skin of mice that rejected MC38-OVA tumors upon Tcm transfer (see Fig. 5a). Pool of two independent experiments represented as individual data and mean ± s.e.m. (n=5/group)

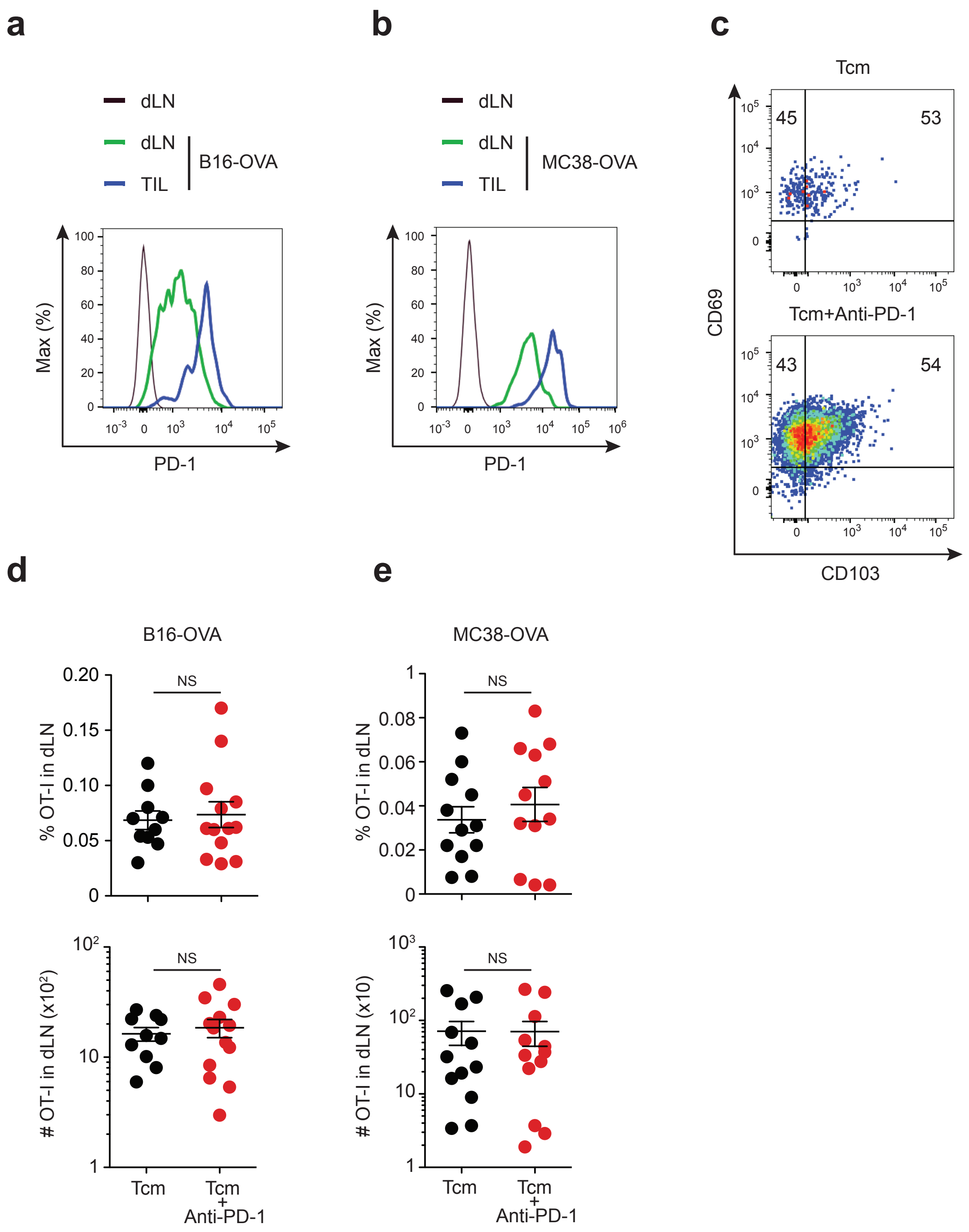

Supplementary Figure 3. PD-1 expression and treatment with anti-PD-1 after Tcm transfer
(**a, b**) Mice were inoculated with B16-OVA i.d. (**a**) or MC38-OVA s.c. (**b**) and OT-I CD45.1^{+} Tcm cells transferred i.v after 7 or 15 d respectively. At day 7 after inoculation of Tcm cells, PD-1 expression in transferred OT-I within the tumor or in the tumor dLN was analyzed. (**c**) Representative FACS dot-plots of tumor infiltrating OT-I cells in 15 mg of B16-OVA tumor. (**d, e**) Mice were inoculated with B16-OVA i.d. (**d**) or MC38-OVA s.c. (**e**), transferred with OT-I Tcm cells and treated with anti-PD1 as indicated in Fig. 6c. Frequency (top) and numbers (bottom) of OT-I cells in dLN are shown. (**a**, **b**) Representative histograms from three independent experiments (n=2-3/control group, 3-4/tumor groups). (**d**, **e**) Pool of three independent experiments represented as individual points and mean ± s.e.m. (n=3-5/group). NS, not significant by two-tailed unpaired Student's t test (**d, e** top) and two-tailed nonparametric Mann-Whitney test (**e** bottom).
